# Conference Reports: NINTH CONFERENCE ON ROOFING TECHNOLOGY National Institute of Standards and Technology, May 4–5, 1989

**DOI:** 10.6028/jres.094.031

**Published:** 1989

**Authors:** Walter J. Rossiter

**Affiliations:** Building Materials Division, Center for Building Technology, National Institute of Standards and Technology, Gaithersburg, MD 20899

The National Institute of Standards and Technology (NIST) Center for Building Technology (CBT) and the National Roofing Contractors Association (NRCA) have joined in sponsoring Conferences on Roofing Technology on a biennial basis since 1969. In 1977, NIST and NRCA co-sponsored the First International Symposium on Roofing Technology, and in 1985, they were joined by RILEM^1^ in co-sponsoring the Second International Symposium on Roofing Technology. These Conferences are the major industry forum for the discussion and dissemination of research results and the presentation of the latest advances in roofing technology. Papers in the Conferences and Symposia address roof performance from fundamental and practical viewpoints.

## 1. The Conference

The theme of the 1989 Conference was “Putting Roofing Technology to Work.” This was appropriate considering the general challenge facing U.S. industry to use our strong scientific and technological capability for making the Nation more competitive in international markets and to build strong links between the base of scientific knowledge and the transfer of knowledge to practice.

Approximately 380 individuals were in attendance. They represented a broad spectrum of the roofing community including architects, engineers, consultants, contractors, specifiers, researchers, and building owners. Fourteen invited papers were presented in a format that included an introductory session and three technical sessions entitled Maintenance, Insulation and Vapor Retarders, and Membranes. An extended question and answer period followed each of the technical sessions with the speakers responding to written questions submitted by the audience and asked by the session chairperson. A Proceedings was published and is available from the NRCA.^2^

## 2. Introductory Session

In this session, William Cullen, formerly of NIST, presented a paper [1] entitled “Transitions in Roofing Technology,” which set the theme for the subsequent presentations. Cullen reviewed the major milestones that the roofing industry has experienced over the last century. His emphasis was on the lessons that have been learned in using technology to solve specific problems. He reminded the audience that technological advances are not always without penalty. He gave several examples where solutions to problems have, at the same time, created new problems that also required technical remedies. He discussed in some detail the advances that have occurred over the last two decades in applying measurement-related technologies to improving roofing performance. His closing comments indicated that, although significant advances have been made, much remains to be done. For example, the application of simulation modeling and use of expert systems are in their infancy in the industry.

## 3. Session on Maintenance

The first two papers [2,3] in the session focused on planned maintenance for extending the life of existing roofing systems, and setting priorities for conducting maintenance activities in cases where a number of roofs are under the responsibility of a single building owner. Owners have considerable investments in roofs, and sound maintenance programs are a means of protecting the investments. The U.S. Army Construction Engineering Research Laboratory (CERL) has developed a systematic approach for assessing the maintenance needs for built-up roofing systems on Army facilities. It is a computerized methodology, called “ROOFER,” that provides an assessment of the condition of a roof based on visual observations from an on-site inspection and tests conducted to determine the amount of moisture in thermal insulation. ROOFER will provide building managers with a procedure for identifying roof problems, and for making practical decisions in setting maintenance and repair strategies and priorities. The program and users’ manuals are expected to be available later in 1989.

The third paper [4] in the session described the U.S. Air Force’s philosophy in obtaining maintainable roofing systems. The requirements consider all parties involved in the service cycle of the roof and include:

- concentrating on maintainability during the design stage,
- obtaining the best quality systems that are available,
- using trained, qualified installers, and
- commitment by the owner to a rigorous maintenance program.

The paper emphasized the last parameter as an item often overlooked by myopic owners who ignore their roles in maintaining roofs. It was pointed out that owners should not expect satisfactory long-term performance from their roofs unless they are willing to invest in a maintenance program.

In concluding the session [5], the latest developments in roof coatings were described. The application of coatings to the surfaces of existing roofs is a maintenance technique that may be used for increasing the longevity of some types of roofing systems. The topics addressed in the paper included a review of the types of coatings available, their uses, advantages, and limitations.

## 4. Session on Insulation and Vapor Retarders

A key parameter affecting the performance of low-sloped roofing systems is the compatibility of the insulation and the membrane. A paper [6] on this subject gave many examples of problems that have arisen when the designer has ignored compatibility. It was pointed out that a database on the performance requirements of insulation/membrane systems has not been developed by the industry. Such a database is needed to establish compatibility requirements.

The U.S. Army Cold Regions Research and Engineering Laboratory (CRREL) has been studying the needs for vapor retarders in low-sloped roofs for many years. The second paper [7] in the session summarized the results of the studies. Maps and graphs that may be used to determine when and where vapor retarders should be installed were presented. The author emphasized the importance of air leakage as a primary mechanism behind condensation problems in buildings, and recommended that the first defense against condensation in low-sloped roofs is to control air leakage into the system.

A major item of concern facing the industry in recent years has been the wind resistance of mechanically fastened single-ply systems. In these systems, the fasteners are normally located in the membrane seams and are driven through insulation boards and attached to the structural deck. Experience has shown that some of these systems have performed less than satisfactorily due to severe wind exposure. Factors affecting their performance are not completely understood. A paper [8] was presented describing the results of tests conducted using a fatigue device developed to simulate wind-uplift conditions. Variables in the testing included the membrane, type and density of insulation, and the type of fastener system and seam. Based on the results of the study, a number of practical recommendations were provided for improving the performance of mechanically fastened systems on exposure to severe wind cycling.

The final paper [9] in the session described the results of an initial experiment on thermal performance using the recently constructed large-scale climate simulator (LSCS) at the Oak Ridge National Laboratory (ORNL). The LSCS provides for thermal measurements of roofing sections subjected to a wide variety of external and internal environments. In the experiment, the thermal resistances of three common insulation boards were measured over an extended range of temperatures using two different techniques, one steady state and the other transient. Results from the two techniques were in agreement with one another and within 5% of reference values published for the test specimens. The initial experiment demonstrated the successful use of the LSCS, thus setting the stage for much needed future research on the thermal performance of low-sloped systems.

## 5. Session on Membranes

The lead paper [10] in this session addressed the application of thermal analysis techniques such as thermogravimetric, dynamic mechanical, and torsion pendulum analyses to the characterization of roof membranes upon aging. A unique aspect of this paper was its co-presentation by an American and a Swiss. The authors had co-chaired a task group under the auspices of the Joint CIB^3^/RILEM International Committee on Elastomeric, Thermoplastic, and Modified Bituminous Roofing. The paper was a synopsis of their task group’s work. In introducing their subject, the authors reminded their audience that the thermal analysis techniques have been long available in many areas of science and technology, but have had little use in assessing roofing membranes, in spite of the sensitivity and reproducibility of the methods. As examples of the utility of the techniques, they presented data taken of rubber, thermoplastic, and polymer-modified bituminous materials before and after exposure to either natural or artificial environments. They concluded by recommending that the use of the methods be refined and standardized specifically for application to roofing membrane materials.

Two papers [11, 13] in the session from NIST addressed the effect of surface contaminants on the strength of adhesive-bonded joints in EPDM single-ply roofing membranes. The first was a laboratory evaluation whereby a known quantity of talc was deposited on cleaned EPDM rubber, and contaminated joint specimens were subsequently prepared. The quantification of the amount of talc involved the use of a computer image analysis method, developed specifically for the study, that was based on the reflectance of light from the “talc-ed” rubber surface. As the amount of talc increased, the surface reflectance of the sheet also increased. The results of the study showed that, for the contaminated T-peel joint specimens, their strength after cure times greater than 2h was highly dependent on the contamination level. The maximum strengths were achieved with specimens that were thoroughly cleaned. In a related NIST field study, strength data were presented for EPDM joint specimens taken from roofs in service. It was found that their strengths were low in comparison to values obtained from control specimens fabricated in the laboratory using cleaned rubber. Scanning electron microscopy of the rubber surfaces of the field specimens showed the presence of talc-like particles that could have contributed to the observed low strengths.

Another paper [12] on membranes discussed the field application of polymer-modified bituminous membranes. The use of these materials has increased substantially in the last few years, and now accounts for about 18% of the current membrane market. The author of the paper reviewed concerns that the industry faces to assure their proper application. Chief among them is the adhesion of the membrane sheets to the roof, as well as to themselves, to form watertight seams. Current adhesion techniques involve thermal methods whereby either heat is applied directly to the sheet to soften the bitumen for bonding, or hot asphalt is used as a bonding agent. Consequently, adequate temperature control under the variety of weather conditions encountered during field application is necessary for proper bonding. To overcome the limitations imposed by the need for precise temperature control, the use of cold-process adhesives for bonding sheets and seams is expected to increase in the future.

The final paper [14] of the Conference was a review of the advances in built-up roofing technology made over the last one and one half decades. Significant developments have occurred such as the development of membrane performance criteria and viscosity-related criteria for asphalt application. The author of the paper expects continued advances with the introduction of new reinforcing fabrics, and polymer-modified asphalts. Reliable techniques for assessing long-term performance will have to be developed to facilitate acceptance of new materials and practices.

## 6. Summary

The papers presented by the authors described problems and concerns that have confronted the roofing industry in recent years. Recommendations were presented for alleviating these problems and providing improvements in roof performance. Nevertheless, in many papers, the authors noted that much remains to be accomplished through research and technology for the continued betterment of the industry. From the perspective of a NIST attendee, it was clear that measurement-related advances in the industry are needed to assist in “putting technology to work,” as the theme of the Conference urged. As examples, the development of methods for assessing long-term performance of new materials, the application of nondestructive techniques for evaluating existing systems and components, and the development of reliable methods for assuring the quality of systems that are fabricated in the field are noted.

## Footnotes

1 Reunion Internationale des Laboratoires d’Essais et de Recherches sur les Materiaux et les Constructions.

2 One O’Hare Centre, 6250 River Road, Rosemont, IL 60018; telephone 312-318-6722.

3 A French acronym indicating the Conseil Internationale due Batiment pour la Recherche, l’Etude, et la Documentation.
